# Validating the Usage of Cariogram in 5- and 12-year-old School-going Children in Paonta Sahib, Himachal Pradesh, India: A 12-month Prospective Study

**DOI:** 10.5005/jp-journals-10005-1495

**Published:** 2018-04-01

**Authors:** Anu Garg, Manish Madan, Parminder Dua, Sheeba Saini, Ritu Mangla, Pallav Singhal, Akash Dupper

**Affiliations:** 1 Postgraduate Student, Department of Pedodontics and Preventive Dentistry Himachal Institute of Dental Sciences, Paonta Sahib, Himachal Pradesh, India; 2 Professor and Head, Department of Pedodontics and Preventive Dentistry Himachal Institute of Dental Sciences, Paonta Sahib, Himachal Pradesh, India; 3 Professor, Department of Pedodontics and Preventive Dentistry Himachal Institute of Dental Sciences, Paonta Sahib, Himachal Pradesh, India; 4 Redear, Department of Pedodontics and Preventive Dentistry Himachal Institute of Dental Sciences, Paonta Sahib, Himachal Pradesh, India; 5 Senior Lecturer, Department of Pedodontics and Preventive Dentistry Himachal Institute of Dental Sciences, Paonta Sahib, Himachal Pradesh, India; 6 Senior Lecturer, Department of Oral Pathology, Sarjug Dental College & Hospital, Darbhanga, Bihar, India; 7 Reader, Department of Conservative Dentistry and Endodontics Yamuna Institute of Dental Sciences & Research, Yamunanagar Haryana, India

**Keywords:** Cariogram, High-risk patients, Low-risk patients, Medium-risk patients.

## Abstract

**Aim:**

To validate the caries risk profiles in 5- and 12-year-old school-going children and to single out main contributing factor, if any, using cariogram over a period of 1 year.

**Materials and methods:**

A cariogram model was used to create caries risk profiles on 499 children aged 5 and 12 years ±6 months. They were divided into 2 groups. The group I and group II consisted of 250 and 249 children respectively. Re-examination was done after 1 year and caries increment was recorded. The caries risk profiles generated by the cariogram software were compared with caries increment.

**Results:**

Percentage of subject having caries increment in groups I and II in high-, medium-, and low-risk group after 1 year was 66.2, 39.5, and 13%, and 48.5, 27.3, and 13.9% respectively. The mean caries increment after 1 year in groups I and II in high-, medium-, and low-risk patients was 0.96, 0.49, and 0.13, and 0.7, 0.36, and 0.11 respectively. Linear regression analysis showed dental caries, diet content, diet frequency, plaque index, *Streptococcus mutans* count, fluoride, salivary flow rate, and buffer capacity are significantly associated with actual chance to avoid caries.

**Conclusion:**

The risk of developing new carious lesions consistently reduced from high-risk category to low-risk category, reflecting the cariogram ability in accurately estimating future caries. Hence, cariogram can be said to be a useful tool for caries prediction. Initial dental caries came out to be the strongest predictor of future caries.

**How to cite this article:** Garg A, Madan M, Dua P, Saini S, Mangla R, Singhal P, Dupper A. Validating the Usage of Car-iogram in 5- and 12-year-old School-going Children in Paonta Sahib, Himachal Pradesh, India: A 12-month Prospective Study. Int J Clin Pediatr Dent 2018;11(2):110-115.

## INTRODUCTION

Everyone has heard the famous quote by Benjamin Franklin, “An ounce of prevention is worth a pound of cure.” This statement certainly echoes the sentiments of the dentistry profession, which has proudly been one of the most proactive of all health care professions in the area of disease prevention.

For developing appropriate preventive approaches, anticipating utilization patterns and planning effectively for organization and financing of dental resources, the knowledge of oral health status and treatment needs of populations with different characteristics is important.^1^ A key factor for planning any preventive program is to accurately access a person’s risk of developing a disease.^2^

Dental caries is a disease of the mineralized dental tissue with a multi-factorial etiology related to the interactions over time between tooth substance, microorganisms, and dietary carbohydrates producing plaque.^3^

Dental caries and its sequel often involve pain and affect esthetics. Treatment of dental caries is costly in terms of both time and money.^4^ It is prudent to prevent dental caries by applying suitable preventive procedures to avoid complications. In developing countries where there is scarcity of resources, the high-risk individuals should be carefully segregated. The idea of caries risk assessment is to identify persons most likely to develop caries and to provide them proper preventive and inter-ceptive treatment.^5^

To assess the susceptibility of caries, Professor D. Bratthall developed the concept and formula for the car-iogram. The “cariogram” is an interactive PC program for caries risk evaluation. It is a graphical picture illustrating in an interactive way the individual’s/patients’ risk for developing new caries in the future, simultaneously expressing to what extent different etiological factors of caries affect the caries risk for that particular patient. It shows a pie diagram that illustrates a possible overall caries risk scenario in the form of five sectors of following colors: green, dark blue, red, light blue, and yellow indicating the different groups of factors related to dental caries.^5^

- The green sector shows an estimation of the “Actual chance to avoid new cavities.”
- The dark blue sector “Diet” is based on a combination of diet contents and diet frequency.
- The red sector “Bacteria” is based on a combination of amount of plaque and mutans streptococci.
- The light blue sector “Susceptibility” is based on a combination of fluoride program, saliva secretion, and saliva buffer capacity.
- The yellow sector “Circumstances” is based on a combination of past caries experience and related diseases.

The cariogram does not specify a particular number of cavities that will or will not occur in the future.^5^

In the year 1997, it was estimated that 22.7% of Indian population is aged 5 to 14 years. This being a high proportion of the population, the prevalence of dental disease among this age group needs to be assessed.^6^ Hence, our study was planned to validate the caries risk profiles in both deciduous and permanent dentition using cariogram in India.

## MATERIALS AND METHODS

The present study was conducted at the schools of Paonta Sahib, District Sirmour (Himachal Pradesh). Ethical clearance and necessary permissions were obtained from the authorities concerned prior to the start of the study.

A total of 520 school-going children aged 5 years ± 6 months (deciduous dentition) and 12 years ± 6 months (permanent dentition) were randomly selected and divided into two groups:

1. Group I: Deciduous dentition (n = 260)
2. Group II: Permanent dentition (n = 260)

Children aged 5 years ± 6 months with fully erupted deciduous dentition and 12 years ± 6 month-old children with fully erupted permanent dentition who were studying in schools located at Paonta Sahib and were permanent residents of Paonta Sahib were included in the study. Children on regular use of chlorhexidine gluconate etc., over the last 3 months or antibiotics for the last 1 month and those having mixed dentition were excluded from the study.

Caries risk profiles were assessed by the cariogram by putting the parameters in PC software in weighted scores using following criteria: related general disease, diet content, diet frequency, fluoride exposure, caries scores at baseline, plaque scores, *S. mutans*—estimation of levels of *S. mutans* in saliva, salivary secretion rate, and salivary buffer capacity. An optional factor for scoring, i.e., clinical judgment, is also available which gives an opportunity for the examiner to express his/her clinical feelings, if it differs from the program in-built estimation. By default, its value is set to “1.”

A set of questions were asked from the patient or patient’s parents/attendants regarding medical history, method of cleaning of teeth, and brushing frequency. Also, history of use of fluorides including the topical fluoride application and toothpaste used was noted and used for scoring. A 24-hour diet recall was done for all the 520 children selected for the study for both the groups. The diet recall was taken by personal interview of the child/ parents by the investigator following the recollection of intake of anything eaten or drunk in the last 24 hours in a backwardly preceding fashion. After the interview, caries prevalence, decayed, missing, and filled teeth (DMFT) index, and decayed, missing, and filled surfaces (DMFS) index were recorded using the World Health Organization (WHO) standard criteria for oral health surveys. Oral hygiene was estimated by employing plaque index. Paraffin-stimulated whole saliva was collected from all the children to measure the (1) saliva secretion rate (expressed as mL/min), (2) buffering capacity of saliva, and (3) *Lactobacillus* and *S. mutans* counts. For *S. mutans,* mitis salivarius-bacitracin agar (HI MEDIA, code 259) was used and for *Lactobacillus,* de Man, Rogosa and Sharpe agar (TM MEDIA, code M641) was used.

These risk factors for dental caries were evaluated using cariogram for both the age groups at baseline. All the children were cariographed to know their caries risk profile i.e., high-risk group, medium-risk group, and low-risk group, which was further kept for record and analysis. At baseline, no intervention or preventive interception was done intentionally. However, children were free to get any knowledge or treatment on their own.

After a period of 1 year, only 499 children were available, i.e., 5 years (n = 250) and 12 years (n = 249), who were considered for statistical analysis.

All the children were reexamined by the same examiner in order to avoid interexaminer variation and the caries status was recorded using DMFT/dmft by WHO criteria. The difference between the caries status at baseline and after one year was assessed and statistical analysis was done.

The results obtained after 1 year were compared with predicted results of cariogram to check the validity of cariogram.

**Graph 1:**
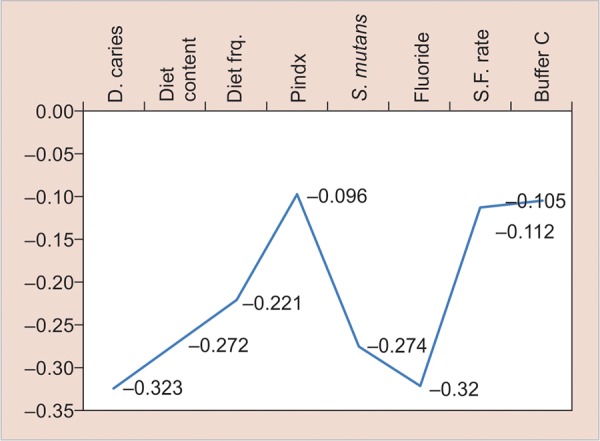
Linear regression analysis of predictor variables in relation to estimation of the “actual chance to avoid new cavities” in group I

**Graph 2:**
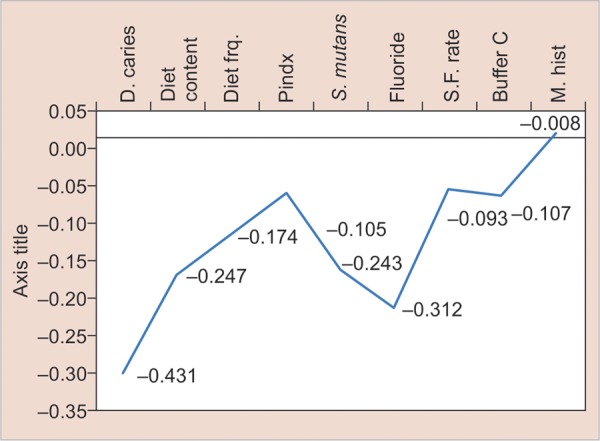
Linear regression analysis of predictor variables in relation to estimation of the “actual chance to avoid new cavities” in group II

### Statistical Methods

The nine factors of the cariogram were compared with actual chance to avoid caries by linear regression analysis. Mean and standard deviation of dental caries increment were calculated after 1-year follow-up according to age and caries risk profile of the cariogram.

## RESULTS

There were total 520 children at the start of the study, divided into two groups of 5- and 12-year-olds (260 each). Out of 520 children, 499 children were available for follow-up after 1 year (21 dropouts) and were considered and examined for the study. The groups are: group I: 5-year-old children—250; group II: 12-year-old children—249.

Linear regression analysis of 5- and 12-year-old children (Graphs 1 and 2) showed that dental caries, diet content, diet frequency, plaque index, *S. mutans,* fluoride, salivary flow rate, and buffer capacity were significantly associated with actual chance to avoid caries. Graph 2 shows that medical history/related disease was statistically not significantly associated with actual chance to avoid caries.

**Graph 3:**
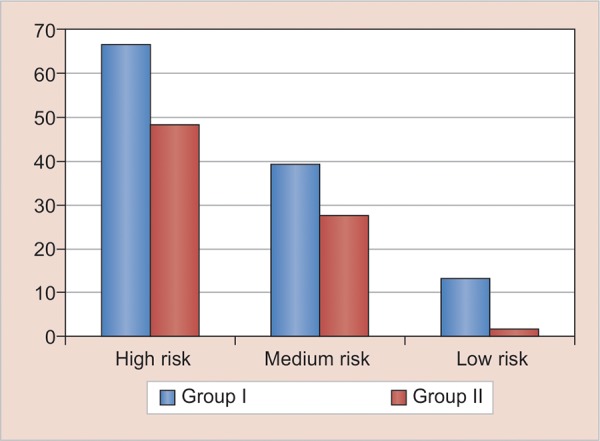
Percentage of subjects having dental caries increment after 1-year follow-up in groups I and II according to caries risk profile of cariogram

**Graph 4:**
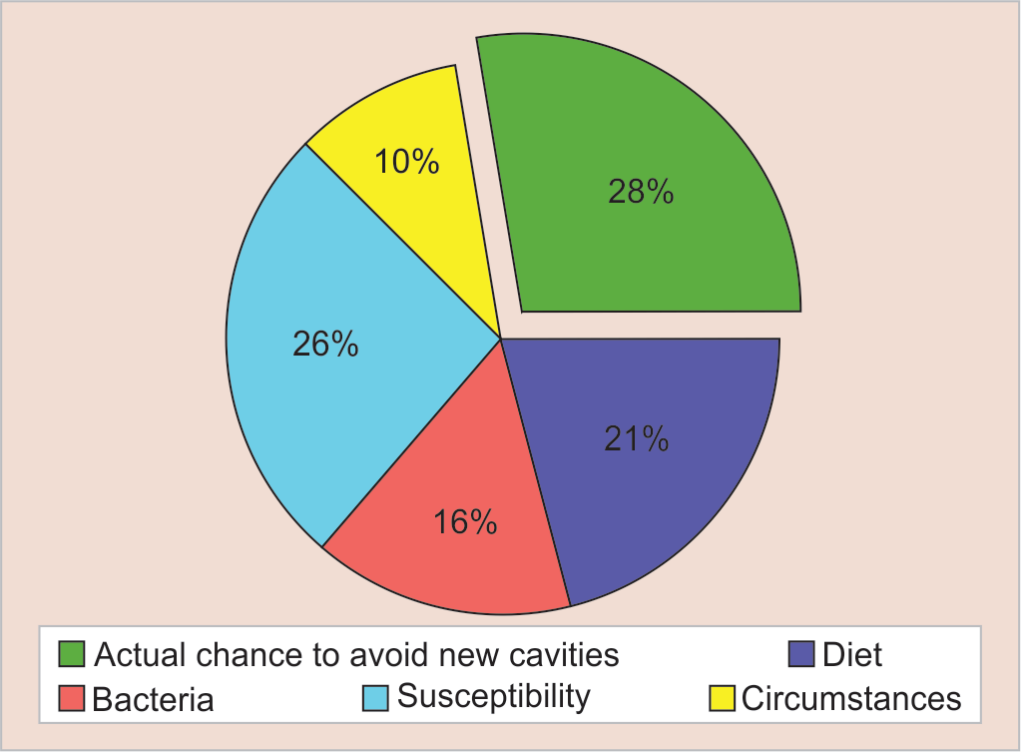
Cariogram group I (5-year-old children) high risk (PC software, version 3.0)

The percentages of subjects having caries increment in group I, i.e., 5-year-old children in high-, medium-, and low-risk groups after 1 year were 66.2, 39.5, and 13% respectively, whereas in 12-year-old children, the percentages of subjects having caries increment were 48.5, 27.3, and 13.9% respectively (Graph 3). Cariogram of a 5-year old child (Group I) has been illustrated in the form of pie diagram which shows that the child is included in high risk group (Graph 4).

Mean caries increment (Table 1) after 1 year in 5-year-old children in high-, medium-, and low-risk patients is 0.96, 0.49, and 0.13 respectively, whereas in 12-year-old children in high-, medium-, and low-risk patients, it is 0.7, 0.36, and 0.11 respectively. The mean caries increment after 1 year in 5-year-old children is higher than in 12-year-old children.

**Table 1:** **Table 1:** Mean and standard deviation of dental caries increment after a 1-year follow-up according to age and caries risk profile of cariogram

*Age group*		*Group*				*n*		*Mean*		*Standard deviation*	
5		Low		New caries		100		0.13		0.338	
		Medium		New caries		76		0.49		0.663	
		High		New caries		74		0.96		0.801	
12		Low		New caries		95		0.11		0.346	
		Medium		New caries		88		0.36		0.647	
		High		New caries		66		0.7		0.803	

## DISCUSSION

The most important challenge in pediatric dentistry is to maintain healthy teeth, and reliable caries risk assessment is essential for early oral health promotion and disease management.^7^ Thus, our study aimed to evaluate the individual caries risk profile of children aged 5 and 12 years. The age groups of 5 and 12 years were chosen in the study, as they are WHO global monitoring ages for dental caries.

Table 1 and Graph 3 are most important with respect to the objective of the study. Table 1 shows that there is an increase in the follow-up mean caries values compared with baseline values. This could be due to the fact that dental caries is a disease with a progressive character, the prevalence of which increases with age in any population independent of sex, urbanization, and social status, probably due to longer exposure time of the dentition to the etiologic factors of caries. It also shows that mean caries increment is higher in children who are at high risk according to cariogram and low in those children who are at low risk according to cariogram. Brathall and Petersson^8^ also showed that the highest-risk group developed almost 10 times more caries compared with the best group (increment 2.58 *vs* 0.27). Similar relationship is seen in other studies.^9,10^

According to Graph 3, highest percentages of individuals developing new caries lesions were observed in children at high risk, and lowest percentages of individuals to develop new lesions were observed in children at low risk according to cariogram in both the groups. The risk of developing new carious lesions consistently reduced from high-risk category to low-risk category, showing the ability of the cariogram in accurately estimating future caries. Hence, a cariogram can be said to be a useful tool for caries prediction. Kemparaj et al^9^ also showed highest percentage of individuals (83.2%) developing new caries lesion in the category of very high-risk children and lowest percentage of individuals (10.8%) to develop new caries lesion in the category of very low-risk children. Petersson et al^11^ also showed in his study that those children with increased risk compared with baseline developed significantly more caries than those with unchanged category.

Linear regression analysis of 5- and 12-year-old children (Graphs 1 and 2) showed that dental caries, diet content, diet frequency, plaque index, *S. mutans,* fluoride, salivary flow rate, and buffer capacity were significantly associated with actual chance to avoid caries.

Caries status was determined by DMFT/dmft.^12^ In our study (Graphs 1 and 2), initial dental caries came out to be the strongest predictor of dental caries in future which is in accordance with the study undertaken by Seppa and Hausen,^13^ in which he showed that caries prevalence in primary teeth can correctly predict future caries in permanent teeth. Celik et al^2^ and Powell^14^ also concluded the same. Sheiham,^15^ Alaluusua et al,^16^ Russel et al^17^ in their epidemiological studies also showed a positive correlation between past caries experience and future caries development. They also stated that initial lesions are supposed to give a better predictability than the number of filled or carious surfaces.

Imfeld,^18^ Midda and Konig,^19^ Woodward and Walker^20^ reported that diet is a major factor influencing dental caries and its role in the caries process is primarily local rather than systemic. In our study, diet factor was judged under 2 parameters: firstly, diet content, i.e., the type of diet individual is consuming (whether low or high fermentable carbohydrates) and secondly, the diet frequency, i.e., the number of meals consumed per day by the child including snacks. In our study, a highly significant correlation was found between the diet content and actual chance to avoid caries (Graphs 1 and 2), which is in accordance with the studies done by Hebbal et al,^4^ and Giacaman et al.^21^

The primary evidence for the belief that the prevalence of dental caries is directly related to the frequency with which sugar is consumed comes from the Vipeholm study.^22^ In our study, we decided to perform a 24-hour diet recall to evaluate the diet frequency of children over a 7-day recall, as it has been reported that a 24-hour recall compared with a 7-day diet record gave comparable mean intakes.^23^ In the present study (Graphs 1 and 2), a highly significant correlation was found between diet frequency and actual chance to avoid caries in both the groups. Celik et al,^2^ Hebbal et al,^4^ Stecksen-Blicks et al,^24^ and Petersson et al^11^ also showed similar results.

Undoubtedly, the most important environmental factor associated with caries is past and present exposure to fluoride, systemic, topical or both, which has consistently shown an inverse relationship with caries prevalence.^25^ In our study, a highly significant positive correlation was found between fluoride program and actual chance to avoid caries, which is in accordance with the studies done by Hebbal et al,^4^ Hanganu and Murariu,^26^ and Peker et al.^27^

Morinushi et al^28^ demonstrated that poor oral hygiene is directly associated with plaque score and contributes to the high prevalence of dental caries. In the present study, evaluation of plaque amount was done using Silness and Loe criteria (1964) and statistically significant difference was found between the plaque index score and actual chance to avoid caries (Graphs 1 and 2). Similar results have been seen in studies done by Celik et al,^2^ Patil et al,^29^ and Hebbal et al.^4^

It is generally accepted that *S. mutans* is the primary causative agent of dental caries in humans.^30,31^ Many studies had reported the correlation between high dental caries experience scores and the presence of high *S. mutans* scores. Toddlers who have high mutans streptococci count in their mouth at 2 and 3 years of age show a noticeably higher risk of developing caries on primary teeth compared with the ones with a low count.^16,32-34^ In the present study, statistically highly significant relation was found (Graphs 1 and 2) between *S. mutans* count and actual chance to avoid caries. Similar results have been found in studies done by Hanganu and Murariu,^26^ Hebbal et al (2012),^4^ Petersson et al,^11^ and Peker et al.^27^

Saliva plays an important role in oral health, as it maintains the integrity of the oral hard and soft tissues, protects the oral tissue against immunologic bacterial, fungal, and viral infections.^35^ In the present study (Graphs 1 and 2), statistically significant relation was found between salivary flow rate and actual chance to avoid caries. Kuriakose et al^36^ and Animireddy et al^37^ also gave similar results in their study.

The buffer capacity of a sample of saliva was measured as the “amount of acid needed to lower the pH of saliva through a fixed pH interval” as is described in Federation Dentaire Internationale technical report no. 31.^36^ A clear inverse relationship between salivary buffer capacity and caries susceptibility has been clearly demonstrated.^36^ In our study (Graphs 1 and 2), a highly significant relation was found between salivary buffer capacity and actual chance to avoid caries, which is in accordance with the studies done by Kuriakose et al^36^ and Animireddy et al.^37^

Related general disease and medical health of each child were taken into consideration, as it has been reported that oral health may be related to some systemic disease.^38^ Graph 2 shows that medical history/related disease was significantly not related to the actual chance to avoid caries. This could be due to the fact that there was just one student with positive medical history.

## CONCLUSION

Dental caries has a high prevalence rate and in a developing country with limited resources, the value of prevention based on caries risk assessment cannot be overemphasized. For a practicing dentist in an industrialized country, cariogram can be used in the daily clinical routine, as it is affordable, user-friendly, and easy to understand. Thus, cariogram can be an effective tool for motivating the patient and also serve as a support for clinical decision making, when selecting preventive strategies for the patient.
